# GCN5 mediates DNA-PKcs crotonylation for DNA double-strand break repair and determining cancer radiosensitivity

**DOI:** 10.1038/s41416-024-02636-4

**Published:** 2024-04-04

**Authors:** Yang Han, Hongling Zhao, Gang Li, Jin Jia, Hejiang Guo, Jinpeng Tan, Xingyao Sun, Saiyu Li, Qian Ran, Chenjun Bai, Yongqing Gu, ZhongJun Li, Hua Guan, Shanshan Gao, Ping-Kun Zhou

**Affiliations:** 1 Department of Radiation Biology, Beijing Key Laboratory for Radiobiology, Beijing Institute of Radiation Medicine, Beijing, China; 2 School of Public Health, Institute for Environmental Medicine and Radiation Hygiene, University of South China, Hengyang, China; 3 Department of Hospital Infection Control, Shenzhen Luohu Peoples Hospital, Shenzhen, China; 4 School of Medicine, University of South China, Hengyang, China; 5 School of life Sciences, Hebei University, Baoding, China; 6 Laboratory of Radiation Biology, Laboratory Medicine Center, Department of Blood Transfusion, The Second Affiliated Hospital, Army Military Medical University, Chongqing, China

**Keywords:** Post-translational modifications, Genomic instability, DNA

## Abstract

**Background:**

DNA double-strand break (DSB) induction and repair are important events for determining cell survival and the outcome of cancer radiotherapy. The DNA-dependent protein kinase (DNA-PK) complex functions at the apex of DSBs repair, and its assembly and activity are strictly regulated by post-translation modifications (PTMs)-associated interactions. However, the PTMs of the catalytic subunit DNA-PKcs and how they affect DNA-PKcs’s functions are not fully understood.

**Methods:**

Mass spectrometry analyses were performed to identify the crotonylation sites of DNA-PKcs in response to γ-ray irradiation. Co-immunoprecipitation (Co-IP), western blotting, in vitro crotonylation assays, laser microirradiation assays, in vitro DNA binding assays, in vitro DNA-PK assembly assays and IF assays were employed to confirm the crotonylation, identify the crotonylase and decrotonylase, and elucidate how crotonylation regulates the activity and function of DNA-PKcs. Subcutaneous xenografts of human HeLa GCN5 WT or HeLa GCN5 siRNA cells in BALB/c nude mice were generated and utilized to assess tumor proliferation in vivo after radiotherapy.

**Results:**

Here, we reveal that K525 is an important site of DNA-PKcs for crotonylation, and whose level is sharply increased by irradiation. The histone acetyltransferase GCN5 functions as the crotonylase for K525-Kcr, while HDAC3 serves as its dedicated decrotonylase. K525 crotonylation enhances DNA binding activity of DNA-PKcs, and facilitates assembly of the DNA-PK complex. Furthermore, GCN5-mediated K525 crotonylation is indispensable for DNA-PKcs autophosphorylation and the repair of double-strand breaks in the NHEJ pathway. GCN5 suppression significantly sensitizes xenograft tumors of mice to radiotherapy.

**Conclusions:**

Our study defines K525 crotonylation of DNA-PKcs is important for the DNA-PK complex assembly and DSBs repair activity via NHEJ pathway. Targeting GCN5-mediated K525 Kcr of DNA-PKcs may be a promising therapeutic strategy for improving the outcome of cancer radiotherapy.

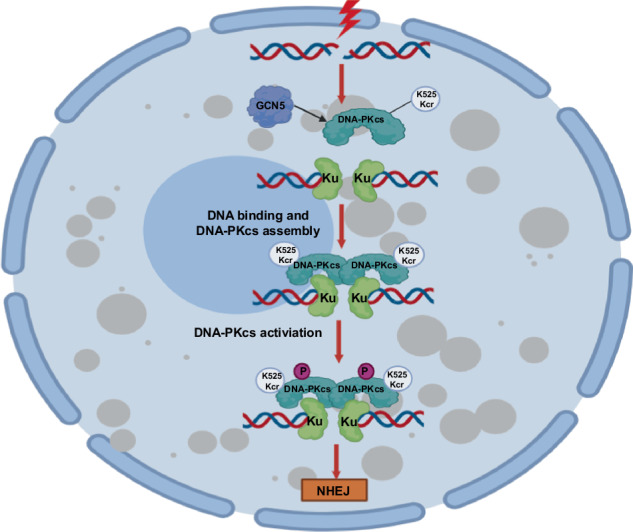

## Introduction

DNA double-strand breaks (DSBs) in DNA are extremely harmful lesions that may lead to gene alterations and cell death if left unrepaired. To maintain the genomic stability, eukaryotic cells generally employ non-homologous end joining (NHEJ) and homologous recombination (HR) pathways to repair DSBs. NHEJ is an error-prone repair pathway in which DSB ends are joined directly without the need for a homologous template, and may cause genomic mutations, such as deletions and insertions [1, 2]. HR is a highly conserved error-free pathway that requires the presence of homologous DNA, and is mainly active in the late S and G2 phases of the cell cycle [3, 4]. The NHEJ pathway is the predominant repair mechanism throughout the cell cycle and is responsible for repairing approximately 80% of all DSBs [5].

As a main DSB repair pathway in mammals, NHEJ is initiated by the KU70/KU80 heterodimer binding to DSBs and the assembly of DNA-PK complex. DNA-PK, consisting of the DNA-PK catalytic subunit (DNA-PKcs) and the KU70/KU80 heterodimer, exerts its influence at the apex of the NHEJ repair pathway [6]. When DSBs occur, the KU70/KU80 heterodimer promptly recognizes and binds to the broken DNA ends within seconds. Subsequently, it recruits DNA-PKcs to the DSB sites via the binding of the carboxyl-terminus of Ku80 with DNA-PKcs amino-terminus [7]. Following this, Artemis nuclease is activated and recruited by DNA-PK to participate in the processing DSBs ends. Finally, assisted by XRCC4 and XLF, DNA ligase IV catalyzes broken DNA end ligation [8, 9]. Remarkably, the assembly of DNA-PK complex is carefully regulated and plays a crucial role in the initiating the NHEJ repair pathway. The interaction of DNA-PKcs with the KU70/80 heterodimer on DNA constitute the central step in the assembly of DNA-PK [10]. However, how DNA-PKcs DNA binding activity is regulated upon DNA damage, and its functions in the assembly of DNA-PK complex and its corresponding kinase activity are still unclear.

DNA-PKcs, a member of the PIKK family, serves as the primary NHEJ kinase responsible for phosphorylating a range of substrates, including H2AX, CHK2, Artemis, 53BP1, Ku, and even itself [11–14]. The DNA-PKcs kinase plays a significant role in DNA damage repair. DNA-PKcs posttranslational modifications can modulate DNA-PKcs autophosphorylation and the interaction of DNA-PKcs with its partners to regulate DNA damage signaling and repair. DNA-PKcs unsergoes auto-phosphorylated or phosphorylated by ATM and ATR at multiple sites within its N-terminal kinase domain. Notably, DNA-PKcs S2056 and T2609 are two major autophosphorylation sites in DNA-PKcs, leading to conformational changes that have a significant impact on the kinase activities and functions during DNA repair [6, 15–17]. Protein phosphatase 6 (PP6) and protein phosphatase 1(PP1) interact and dephosphorylate DNA-PKcs to promote DNA-PKcs activity [18, 19]. In addition to phosphorylation modification, TIP60-mediated acetylation, HUWE1-mediated neddylation and PARP1-associated PARylation also critically affect the regulation and activation of DNA-PKcs [20–22]. SIRT6 stabilizes DNA-PKcs on chromatin while promoting the function of the latter NHEJ [23]. The deacetylation of DNA-PKcs by SIRT2 can active DNA-PKcs, consequently enhancing the interaction between DNA-PKcs and KU70/80 heterodimer [24].

Protein lysine crotonylation (Kcr) is a recently discovered post-translational protein modification found in proteins involved in various biological activities including gene transcription control [25, 26], sperm formation [27, 28] and DNA damage repair [29, 30]. This modification shares several enzymes with acetylation [31]. In a study by Enas R. Abu-Zhayia et al. it was observed that H3K9cr levels transiently decreased after laser microirradiation-induced DNA damage, marking the first indication of Kcr’s potential impact on DDR [30]. CDYL1, which functions as a crotonyl-CoA hydratase, can reduce Kcr level at DSB sites through its crotonyl-CoA hydratase activity. This leads to the expulsion of the transcription elongation factor ENL and subsequent transcriptional silencing [26, 32, 33]. Kcr modifications of RPA1 play a role in promoting the binding of ssDNA and recruiting RPA1 to DNA damage sites in the HR repair pathway [29]. Additionally, H2AK119cr (lysine crotonylation of H2A on lysine 119) is involved in replication stress response [34].

General control nondepressible 5 (GCN5), also called lysine acetyltransferase 2 A (KAT2A), was the initial histone acetyltransferase cloned and characterized in yeast, and it has been shown to exert essential effects on chromatin and epigenetic modifications. GCN5-mediated acetylation modification is associated with numerous biological processes, including chromatin regulation [35], neuronal apoptosis [36], cell proliferation [37], autophagy [38], inflammation [39], stem cell differentiation [40], and the response to oxidative stress response [41]. Furthermore, GCN5-mediated acetylation modifications are linked to DNA damage repair. In the event of DNA damage, GCN5-mediated H3 acetylation collaborates with SWI/SNF to facilitate efficient H2AX phosphorylation. Deletion of GCN5 in cells results in the impairment of the recruitment of several DNA damage repair proteins to DNA damage sites [42]. Acetylation of RPA1 by GCN5 enhances nucleotide excision repair, although it may not be required in other DNA repair pathways in response to UV irradiation [43]. E2F1 has the capability to binds to GCN5 and recruits GCN5 to the sites of UV-induced DNA damage sites [44]. However, its regulatory mechanism and function in DSBs repair and DNA damage induced by IR remain unclear.

The present work illustrated the regulatory mechanism modulating DNA-PK assembly and activation through GCN5-mediated DNA-PKcs K525 crotonylation via promotion of DNA-PKcs binding to DNA and KU70/80 heterodimer, facilitating the recruitment of DNA-PKcs at DSB sites and NHEJ repair. GCN5 promotes the interaction between DNA-PKcs and Ku70/80/DNA complex. Knockdown of GCN5 results in deficiency of DNA-PKcs recruitment and activation, thereby impairing the NEHJ repair and sensitizing tumor cells to DNA damage-inducing agent. In summary, our study elucidates a regulatory mechanism concerning the binding affinity of DNA-PKcs for DNA and its impact on DNA-PK assembly, recruitment, activation, and NHEJ repair following DNA damage. GCN5 emerges as a promising therapeutic target for enhancing the effectiveness of drugs used to induce DNA damage in cancer therapy.

## Methods

### Cell culture and transfection

The cells used for the experiments were obtained from the American Type Culture Collection (ATCC) and including HEK-293T (human embryonic kidney epithelial cell line), U2OS and HeLa cells. The HEK-293T and HeLa cells were cultivated in 10% (v/v) FBS-DMEM (Dulbecco’s modified Eagle’s medium) supplemented with 1% (v/v) penicillin–streptomycin. The U2OS cells were cultivated in 10% (v/v) FBS-McCoy’s 5A supplemented with 1% (v/v) penicillin–streptomycin. Moreover, the cells were maintained at 37 °C with 5% CO_2_. Lipofectamine 2000 (Invitrogen) was utilized for transfection in accordance with the manufacturer’s instructions.

### Mice

Female BALB/c-nu mice aged 4 weeks were obtained from SPF (Beijing) Biotechnology Co. Ltd and were housed in a specific pathogen-free (SPF) animal facility at the experimental animal center of Academy of Military Medical Sciences. The mice have unrestricted access to autoclaved food, bedding, and water. Each cage contained 5–6 mice and was situated in a room with controlled humidity and temperature. Our animal experimental procedures received approval from the Animal Care and Use Committee at the Academy of Military Medical Sciences and were conducted in accordance with the Laboratory Animal Guideline of Welfare and Ethics (GB/T 35892–2018).

### Antibodies and chemical reagents

The antibodies utilized in this study were as follows: Anti-Flag (F3165, Sigma-Aldrich, RRID: AB_259529), Anti-Flag@M2 Affinity Gel(AZ220, Sigma),Anti-GCN5 (sc-365321, Santa Cruz Biotechnology, RRID: AB_10846182), Anti-Crotonyllysine Mouse mAb (PTM-502, PTM BIO, RRID: AB_2877695), Anti-Acetyllysine Rabbit pAb (PTM-105, PTM BIO, RRID: AB_2877698), Anti-GAPDH Rabbit mAb (PTM-5375, PTM BIO), Anti-Myc (16286-1-AP, Proteintech, RRID: AB_11182162), Anti-HDAC3 (A2139, ABclonal, RRID: AB_2764158), Anti-DNA-PKcs (MA5-13238, Thermo scientific, RRID: AB_10988612), Anti-DNA PKcs (phospho S2056) antibody (ab18192, Abcam, RRID: AB_869495), Anti-γ-H2AX (05-636, Millipore, RRID: AB_309864), Anti-Ku70 (sc-17789, Santa cruz, RRID: AB_628454), Anti-Ku80 (sc-9034, Santa cruz, RRID: AB_2218743), Anti-XRCC4(sc-271087, Santa cruz, RRID: AB_10612396), Anti-Artemis (sc-518193, Santa cruz), Anti-Phospho-XRCC4 (Ser260) Antibody (AF8336, Affinity Biosciences, RRID: AB_2840398), Anti-Phospho-Artemis (Ser516) Antibody (347346, Zenbio), Alexa Fluor 488-labeled Goat Anti-Mouse IgG(H + L) (A-21202, Invitrogen, RRID: AB_141607), Alexa Fluor 568-labeled Goat Anti- Rabbit IgG(H + L) (A-11036, Invitrogen, RRID: AB_10563566). We obtained the Annexin V, FITC Apoptosis Detection Kit from Dojindo, QuickMutation™ Plus Kit(D0208S), and the GST-tag Protein Purification Kit (P2262) were purchase from Beyotime, Hydroxyurea (400046-5GM), Etoposide (E1383), Campathecin (C9911), and Mitomycin C (M0503) were obtained from Sigma-Aldrich. TSA (S1045) and NAM (S1899) were purchase from Selleck. PI/RNase Staning Solution (CY001-L) was purchase from SIMUBIOTECH. LightShift™ Chemiluminescent EMSA Kit (20148) and Hoechst 33342 (62249) were purchased from ThermoFisher Scientific. EpiMark® Nucleosome Assembly Kit (E5350S) was purchased from New England BioLabs. DAPI (ZLI-9557) was purchased from ZSGB-BIO. DH5α(TSC-C14) and BL21(TSC-E01) were purchased from Tsingke Bio. Crotonoyl coenzyme A (28007-5MG) was  purchased from Sigma.

### Plasmids and siRNAs

Flag-tag plasmids including CBP, P300, GCN5, PCAF, and SFB-TIP60 plasmid were gifted from Jiadong Wang’s laboratory (Peking University Health Science Center, Beijing, China). Myc-tagged-HAC1, HDAC3, and HDAC8 plasmids were generated by PCR amplification prior to their insertion into the pcDNA3.1-Myc vector. Flag-DNA-PKcs truncation mutant plasmids were constructed in our laboratory. Flag-DNA-PKcs truncation mutant B K525R, Flag-DNA-PKcs truncation mutant B K525N, GCN5 siRNA1–resistant mutants, and HDAC3 siRNA1–resistant mutants were generated by using the QuickMutation™ Plus Kit following the provided instructions, and their sequences were confirmed through sequencing. The GST-DNA-PKcs truncation mutant B WT, GST-DNA-PKcs truncation mutant B K525R and GST-DNA-PKcs truncation mutant B K525N cDNAs were inserted into GST-pET-4T-1 to achieve GST-tag expression. GST-HDAC3, GST-GCN5, GST-Ku70, and GST-Ku80 were inserted into GST-pET-4T-1 to achieve GST-tagged expression. Full-length Ku70 and Ku80 cDNAs were cloned and inserted into the pcDNA-3.1- GFP vector.

siRNAs were prepared by GenePharma Biotech (Shanghai). During transfection, cells were transfected with specific siRNAs twice using Lipofectamine 2000 (Invitrogen, 11668027) and separated for 24 h following the specific instructions. The siRNA sequences used are shown below:

GCN5-siRNA1: CCCUGGAGAAGUUCUUCUATT;

GCN5-siRNA2: CAGCCCUCCAUUUGAGAAATT;

HDAC3-siRNA1: CAACAAGAUCUGUGAUAUUUU;

HDAC3-siRNA2: CUGACAAUGGUACCUAUUAUU;

53BP1-siRNA: GAGAGCAGAUGAUCCUUUAdTdT;

BRCA1-siRNA: CAGCUACCCUUCCAUCAUAUUdTdT.

### Co-IP and Western blotting

For the Co-IP assay, cells were lysed in NETN buffer (containing 300 mM NaCl, 20 mM Tris-base, 1 mM EDTA, and 0.5% (v/v) NP-40) supplemented with a protease inhibitor cocktail (cOmplete). After 20 min of continuous agitation at 4 °C, cell lysates were centrifuged at 12,000 rpm for 10 min to collected the supernatant, which was then incubated with beads or antibodies at 4 °C for 6 h. Afterward, the samples were rinsed thrice with NETN buffer supplemented with protease inhibitors. The eventual immunoprecipitate samples were subjected to SDS–polyacrylamide gel electrophoresis (SDS–PAGE) for separation before antibody incubation. For Western blotting, the cells were lysed in NETN buffer supplemented with protease inhibitor cocktail (cOmplete). Cell lysates were obtained after 20 min of constant agitation at 4 °C and centrifuged at 12,000 rpm for a 10-min period. Following separation by SDS-PAGE, the final samples were examined using the appropriate antibodies.

### In vitro crotonylation and decrotonylation assay

In vitro crotonylation assay was conducted using 1 µg GST-DNA-PKcs-B WT or K525R, 0.2 µg GCN5, and a mixture containing 300 µM crotonyl-CoA in a 50 µL reaction mixture composed of 50 mM Tris-Cl (pH 7.5), 100 mM NaCl, 1 mM EDTA, and 1 mM DTT). The reaction was carried out at 37 °C for 1 h period. Subsequently, enzyme inactivation was carried out at 70 °C for a duration of 5 min. In addition, 0.2 µg of HDAC3 was mixed with the samples and incubated at 37 °C to achieve decrotonylation. SDS-PAGE was subsequently performed for sample separation, while an anti-Kcr antibody was used for Western Blotting for sample detection.

### Electrophoretic mobility shift assay (EMSA)

The expression and purification of proteins, including DNA-PKcs-B WT and DNA-PKcs-B K525R/N, Ku70, and Ku80, were accomplished in BL21 E. coli. Subsequently, EMSA assays were conducted using the EMSA Kit (Thermo Fisher #20148) following the manufacturer’s instructions. Briefly, proteins at specific concentrations were blended with biotin-labeled DNA (2 fM) substrate in a 20 µL reaction mixture (5 mM MgCl2, 2.5% glycerol, 50 ng/µL poly (dI.dC), 0.05% NP-40). The samples were subsequently subjected to a 20-min incubation at room temperature. Finally, the samples were separated on 6% native polyacrylamide gels in 0.5× TBE buffer (2 mM EDTA, 90 mM boric acid, 90 mM Tris-HCl, pH 8.3) and detected with an HRP-conjugated biotin antibody using a GE ImageQuant™ LAS 500 Imaging System.

### GST pull-down assay

For the DNA-PKcs-B WT/K525N GST pull-down assays, the fusion proteins were expressed and purified in BL21 E. coli by using glutathione Sepharose 4B beads. After purification, 4 µg fusion proteins were subjected to incubation with cell lysates (with or without DNase I treatment) at 4 °C for 2 h followed by mixing with 20 µL of GST-beads incubated after 3 h of incubation at 4 °C. Following that, SDS-PAGE was employed to separate the samples, and Western blotting was carried out for protein analysis.

For GST-DNA-PKcs-B WT/K525N, Ku70, Ku80 in vitro GST pull down assays, the fusion proteins were expressed and purified in BL21 E. coli by using glutathione Sepharose 4B beads. After purification, 2 µg GST-DNA-PKcs-B fusion protein and 20 µg Ku70, Ku80 proteins were mixed with or without biotin-labeled dsDNA (2 fM) in a reaction buffer (2.5% glycerol, 5 mM MgCl2, 50 ng/μL poly (dI.dC)) in a 100 μL system, followed by 20 mins incubation at room temperature for protein-DNA binding. Then, the samples were diluted 10 times with NETN buffer prior to 2 h of incubation at 4 °C for protein binding, followed by mixing with 20 µL GST-beads incubated at 4 °C for 3 h. SDS‒PAGE was used for sample separation, whereas Western blotting was used for protein analysis.

### In vitro DNA-PK assembly assay

With the EpiMark® Nucleosome Assembly Kit (NEB #E5350S), we conducted in vitro DNA-PK assembly assays following the provided instructions. In brief, Ku70, Ku80, DNA-PKcs-B WT and K525N proteins were expressed and purified in BL21 by utilizing glutathione Sepharose 4B beads. After purification, the purified proteins (5 μM) were mixed with biotin-labeled dsDNA (5 μM) in 1 M NaCl at a final volume of 20 μL and Incubate reactions at room temperature for 30 min. Finally, the samples were separated on 6% native polyacrylamide gels in 0.5× TBE buffer (containing 2 mM EDTA, 90 mM Tris-HCl, 90 mM boric acid, pH 8.3) and then detected using an HRP-biotin antibody with the GE ImageQuant™ LAS 500 Imaging System.

### NHEJ DNA repair assay

The efficiencies NHEJ DNA repair were examined via NHEJ assays Briefly, the indicated siRNAs were transfected into HeLa cells that incorporated EJ5-GFP reporters. Then, p-cherry- and I-Scel-expressing vectors were transfected into the cells. DOX was subsequently added to elicit I-SecI expression, and the proportions of GFP- or RFP-positive cells were examined via fluorescence-activated cell sorting (FACS) after 48 h. The proportion of GFP-positive cells among the RFP-positive cells was calculated as the NHEJ repair efficiency, while the repair frequency is presented as the means ± SDs from three or more assays.

### Immunofluorescence assay

Following coverslip culture in dishes (35 mm), treatment of HeLa cells was accomplished using ionizing radiation (IR) at a dose of 4 Gy or not, cells were harvested at the specified time points. Then, the cells were subjected to PBS (phosphate-buffered saline) rinsing, paraformaldehyde (4%) fixation at ambient temperature for 10 min, and subsequently permeabilized with Triton X 100 (0.25%) for an additional 10-min period at room temperature. Then blocked with 5% FBS at room temperature for 1 h and a subsequent 60-min incubation of cells at ambient temperature with corresponding antibodies. Subsequently, the samples were washed and incubated with secondary antibody for 60 min. DAPI staining was performed to visualize nuclear DNA. Finally, coverslips were placed onto slides containing anti-fade buffer, and the results were visualized with a fluorescence microscope (Nikon).

### Mass spectrometry

HeLa protein lysates were used in immunoprecipitation experiments with IgG or DNA-PKcs antibody. DNA-PKcs protein was eluted from beads and subjected to LC–MS/MS analysis. For LC-MS/MS analysis, the tryptic peptides were dissolved in 0.1% formic acid (solvent A), directly loaded onto a home-made reversed-phase analytical column (15 cm length, 75 μm ID). The gradient was comprised of an increase from 6% to 23% solvent B (0.1% formic acid in 98% acetonitrile) over 16 min, 23% to 35% in 8 min and climbing to 80% in 3 min then holding at 80% for the last 3 min, all at a constant flow rate of 400 nl/min on an EASY-nLC 1000 UPLC system. Then peptides were subjected to NSI source followed by tandem mass spectrometry (MS/MS) in Q ExactiveTM Plus (Thermo) coupled online to the UPLC. The electrospray voltage applied was 2.0 kV. The m/z scan range was 350 to 1800 for full scan, and intact peptides were detected in the Orbitrap at a resolution of 70,000. Peptides were then selected for MS/MS using NCE setting as 28 and the fragments were detected in the Orbitrap at a resolution of 17,500. A data-dependent procedure that alternated between one MS scan followed by 20 MS/MS scans with 15.0 s dynamic exclusion. Automatic gain control (AGC) was set at 5 × 10^4^. DNA-PKcs IP and mass spectrometry were conducted in duplicates. The mass spectrometry was completed by JingJie PTM Biolab (Hangzhou) Co. Inc.

### Laser microirradiation assay

HeLa and U2OS cells transfected with indicated plasmids or transected with non-targeting siRNA or GCN5 siRNA. 24 h later, cells were plated into 35-mm glass bottom dishes (MatTek Corporation). Laser microirradiation (UV laser at 365 nm) was performed using the NIKON Ti2 microscope with Ilas Pulse (GATACA SYSTEMS) at a laser output of 7.5%, ensuring the consistent generation of focused GFP stripes. Images were captured at 1 min intervals within a 5 min period following the damage using Micro Manager software. Moreover, Image J software was used to quantify the localization of GFP (the GFP signal at the microirradiation bands), and the relative fluorescence intensity ratio of multiple cells (the irradiated region minus the background) were calculated. For each condition, 10 biological replicates were performed.

### Clonogenic survival assay

For colony formation, HeLa cells were seeded in six-well plates and treated with the indicated doses of irradiation. The cells were incubated in medium and cultured for another 2 weeks. Later, the cells were stained with 0.5% crystal violet in 20% methanol-involving PBS, after which the colonies containing over 50 cells were counted.

### Cell viability assay

HeLa cells (2000/well) were inoculated into 96-well plates, which were subsequently treated with HU (Sigma-Aldrich #400046-5GM; 1 mM), Etoposide (Sigma-Aldrich #E1383; 100 nM), Campathecin (Sigma-Aldrich #C9911; 1 μM) or Mitomycin C (Sigma-Aldrich #M0503; 5 μM). 24 h later, CCK8 regent (Dojindo #CK04) was added to analyze cell viability. The data are presented as the means ± SDs of triplicate experiments (at least).

### Flow cytometry

After trypsinization, the cells were rinsed with prechilled PBS. For analysis of the cell cycle, cells were immersed prechilled 70% ethanol at −20 °C overnight prior to 5 min of centrifugated with 1000 rpm at 4 °C, then the pellets were suspended in 300 μL PI/RNase Staining Solution (SIMUBIOTECH# CY001-L) for 30 min at room temperature. For analysis of apoptosis, the Annexin V, FITC Apoptosis Detection Kit (DOjindo#AD10) was used according to the manufacturer’s instruction. Fluorescence-activated cell sorting (FACS) analysis was performed by flow cytometry, and the percentage of apoptotic cells were recorded using a flow cytometer from FACSCalibur (ACEA Biosciences).

### Tumorigenicity experiment

For all experiments, 4-week-old female BALB/c-nu mice obtained from SPF (Beijing) Biotechnology were used and maintained under SPF conditions. The animals were randomly divided into four groups (5 mice/group): Control siRNA group, GCN5 siRNA group, Control siRNA+ IR group, GCN5 siRNA+ IR group. GCN5 WT or GCN5 knockdown HeLa cells were digested with 0.25% trypsin and washed twice with PBS, and the cell concentration was adjusted to 1 × 10^8^ cells/ml with PBS. Following group assignment, 100 μL cell solution was subcutaneously injected into right axilla of female nude mice (five mice for each group). Three days later, mice were locally irradiated with 10 Gy γ-ray or not. Tumor growth was monitored at 3-day intervals, and the tumor volume was calculated using the formula: tumor volume = length × width^2^/2. The tumors were removed approximately 3 weeks postinjection to determine the tumor weight. All animal experiments were performed following the Laboratory Animal Guideline of Welfare and Ethics (GB/T 35892–2018). Our animal experimental protocols were approved by the Animal Care and Use Committee at the Academy of Military Medical Sciences.

### GST fusion protein purification

To purify GST fusion protein, the instructions provided by the manufacturer of the GST-tagged protein purification kit (Beyotime) were followed. Briefly, GST proteins are produced from 1 L of BL21 competent cells cultured in LB medium. A standard expression induction procedure was performed by shifting log-phase cultures (A600 = ~0.6) from 37 °C and adding 1 mM IPTG. After 16 h with constant vigorous shaking, cells were pulled down by centrifugation at 5000 rpm at 4 °C for 10 min. Pelleted bacterial cells were lysed for 20 min and sonicated, and insoluble molecules were removed. Then, we placed the soluble lysates into a tumbler at 4 °C with BeyoGold™ GST-tag Purification Resin, next the resin was washed with a washing solution, and finally the GST fusion proteins were eluted using an elution solution.

For the purification of the Ku70, Ku80, GCN5 and HDAC3 proteins, GST-Ku70, GST-Ku80, GST-GCN5, and GST-HDAC3 protein solutions were obtained. Following that, PreScission Protease was introduced into the protein solution (at the ratio of 2U PreScission Protease for every 100 μg of GST-tagged protein). The digested protein samples were then added to BeyoGold™ GST-tag Purification Resin that had been pre-equilibrated with PreScission Protease digestion buffer, and bind for 20–30 min at room temperature. Subsequently, supernatants containing the excised GST-tagged target proteins were obtained by centrifugation for 5 min at 500 g. The undigested GST-tagged proteins and PreScission Protease remained bound in the gel precipitate.

### Quantification and statistical analysis

The mRNA-seq data were constructed by exploiting the dataset from TCGA Data Portal (https://tcga-data.nci.nih.gov/tcga/). GCN5 expression in tumor and noncarcinoma tissue samples was examined via Spearman’s test. Kaplan‒Meier analysis was performed for survival analysis. *P < 0.05, according to the log-rank test indicates a significant difference.

Group data were analyzed by two-tailed unpaired Student’s *t* test (GraphPad Prism software, version 8.02) and expressed as means ± SD unless otherwise indicated. *P < 0.05 indicates statistical significance.

## Results

### DNA-PKcs K525 crotonylation is enhanced upon DNA damage

**Fig. 1:**
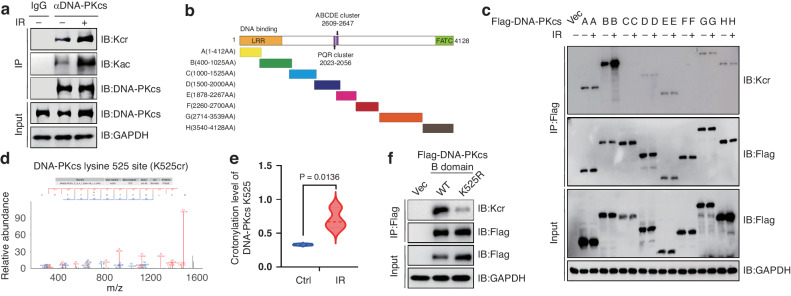
DNA-PKcs crotonylation is enhanced in response to IR-induced DNA damage. **a** HeLa cells were irradiated with or without 8 Gy γ-ray, then DNA-PKcs crotonylation level was analyzed by denaturing pull-down and immunoblotting. **b** The schematic show of different DNA-PKcs truncation mutants. **c** 293 T cells were transfected with indicated plasmids harboring the indicated DNA-PKcs fragments, and then the crotonylation level was analyzed by denaturing pull-down and immunoblotting. **d** Identification of crotonylation sites in DNA-PKcs by high resolution LC-MS/MS. **e** Quantitative changes in DNA-PKcs K525 crotonylation site before and after irradiation according to LC-MS/MS data. **f** 293 T cells were transfected with WT DNA-PKcs B (400–1025aa) or the indicated single point mutant (K525R), the crotonylation levels were detected by denaturing pull-down and immunoblotting.

DNA-PKcs is a pivotal kinase involved in the initiation of DSB repair via the NHEJ pathway, and its activity is regulated by multiple post-translational modifications (PTMs). Crotonylation is a recently identified PTM that has a critical effect on regulation of DSBs repair. To investigate whether DNA-PKcs is modified by crotonylation, Co-IP assay was performed to detect the crotonylation of DNA-PKcs. The result indicated that DNA-PKcs was modified by crotonylation, which is dramatically increased in response to DNA damage. Additionally, lysine acetylation (Kac) of DNA-PKcs was concurrently detected, and the acetylation level of DNA-PKcs increased after irradiation, consistent with previous research (Fig. 1a). To identify the crotonylation sites of DNA-PKcs, the crotonylation of DNA-PKcs truncates were detected. We observed that the most significant crotonylation of DNA-PKcs occurred within the region of 400–1025 aa (the Flag-DNA-PKcs B domain, which is the DNA binding domain) and was enhanced upon DNA damage (Fig. 1b, c). Subsequently, mass spectrometry assay was conducted (supported by JingJie PTM Biolab (Hangzhou) Co. Inc), and the results indicate that the crotonylation at K525 site of DNA-PKcs was significantly increased following exposure to ionizing radiation (IR) (Figs. 1d, e, S1). To further confirm this finding, the lysine residue at position 525 was substituted with arginine, resulting in a substantial reduction in crotonylation compared to the wild-type (WT) (Fig. 1f).

### GCN5 and HDAC3 co-regulate the crotonylation of DNA-PKcs

**Fig. 2:**
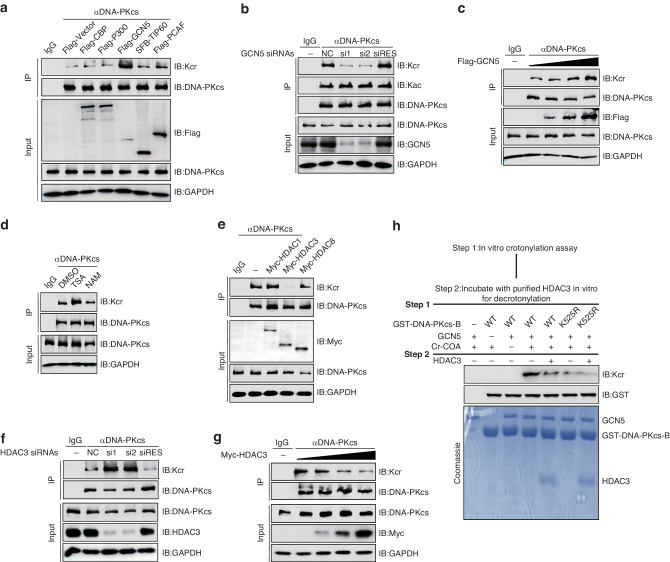
GCN5 and HDAC3 co-regulate the crotonylation of DNA-PKcs. **a** HEK293T cells transiently transfected the indicated plasmids, and then DNA-PKcs crotonylation levels were analyzed by denaturing pull-down and immunoblotting. **b** HeLa cells were transfected with indicated siRNA and plasmids, and then DNA-PKcs crotonylation levels were analyzed by denaturing pull-down and immunoblotting. **c** HEK293T cells transiently transfected indicated plasmids and gradient GCN5 plasmids in concentrations of 0.5 μg, 1 μg, 2 μg, then DNA-PKcs crotonylation levels were analyzed by denaturing pull-down and immunoblotting. **d** HeLa cells were pretreated with 1 μM TSA or 10 mM NAM for 10 h, then DNA-PKcs crotonylation levels were analyzed by denaturing pull-down and immunoblotting. **e** HEK293T cells transiently transfected the indicated plasmids, and then DNA-PKcs crotonylation levels were analyzed by denaturing pull-down and immunoblotting. **f** HeLa cells were transfected with indicated siRNA and plasmids, and then DNA-PKcs crotonylation levels were analyzed by denaturing pull-down and immunoblotting. **g** HDAC3 dose-dependently disrupted DNA-PKcs crotonylation in 293 T cells. **h** GCN5 crotonylated and HDAC3 decrotonylated DNA-PKcs in vitro. Flowchart for the in vitro assay (upper panel). To specify, GST-DNA-PKcs-B WT and its K525R mutant expressed and purified from E. coli were used as substrates, while GCN5 and HDAC3 expressed and purified from E. coli were used as enzymes. The in vitro crotonylation assay was performed, followed by in vitro decrotonylation assay, as described in Materials and Methods. Samples were separated by SDS-PAGE and blotted with indicated antibodies.

Protein crotonylation and acetylation share many common acetylases and deacetylases as indicated in previous reports [31, 45, 46]. To identify the enzyme responsible for crotonylation of DNA-PKcs, a series of acetyltransferases were overexpressed in 293 T cells, followed by immunoprecipitation and western blotting. Consequently, overexpression of GCN5 significantly increased the crotonylation of DNA-PKcs (Fig. 2a). To further confirm that GCN5 is the crotonylase of DNA-PKcs, DNA-PKcs crotonylation were measured in GCN5 knockdown cells. GCN5 knockdown apparently decreased DNA-PKcs crotonylation but not acetylation, which was rescued by transfecting GCN5 siRNA resistant (siRES) plasmid (Fig. 2b). DNA-PKcs crotonylation levels gradually increased with the increasing expression of GCN5 (Fig. 2c). These results demonstrated that GCN5 is the crotonylase of DNA-PKcs.

To identify the decrotonylase of DNA-PKcs, HeLa cells were treated with trichostatin A (TSA, an HDAC-specific inhibitor) or nicotinamide (NAM, a SIRT-specific inhibitor), and then DNA-PKcs crotonylation were detected. As depicted in Fig. 2d, TSA exposure significantly elevated DNA-PKcs crotonylation, whereas NAM treatment had minimal effect on the DNA-PKcs crotonylation. This suggests that the decrotonylase of DNA-PKcs belongs to HDAC family. To further clarify the decrotonylase of DNA-PKcs, different deacetyltransferases of HDAC family were overexpressed in 293 T cells prior to immunoprecipitation and western blotting. HDAC3 overexpression significantly decreased the amount of crotonylation of DNA-PKcs (Fig. 2e). HDAC3 knockdown led to an increase in of DNA-PKcs crotonylation (Fig. 2f). DNA-PKcs crotonylation levels were gradually decreased with increasing HDAC3 expression (Fig. 2g). Therefore, HDAC3 is a specific decrotonylase of DNA-PKcs.

To confirm these results, we further purified the GST-DNA-PKcs B (400–1025 aa) WT and its K525R mutant and assayed crotonylation levels in vitro. As shown in Fig. 2h, DNA-PKcs B WT was crotonylated by GCN5(lane 4), which was subsequently decrotonylated by HDAC3(lane 5). Nevertheless, the K525R mutant was unable to undergo crotonylation by GCN5. Taken together, our results demonstrated that GCN5 and HDAC3 co-regulate DNA-PKcs K525 crotonylation as the crotonylase and decrotonylase, respectively.

### K525 crotonylation promoted the recruitment of DNA-PKcs to DNA damage sites

**Fig. 3:**
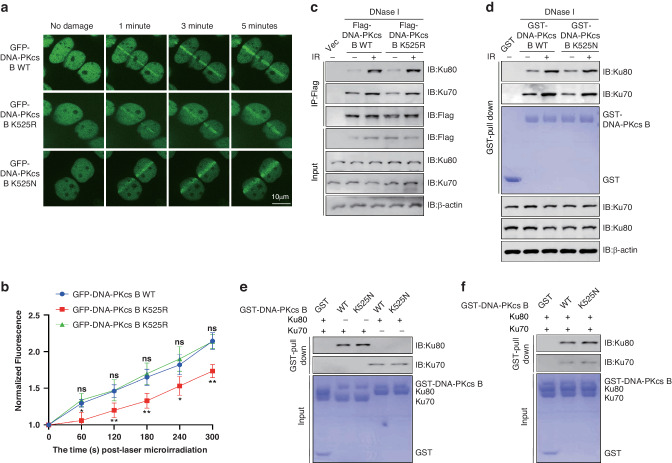
K525 crotonylation promoted the recruitment of DNA-PKcs to DNA damage sites. **a** Laser microirradiation was conducted on U2OS cells transfected with GFP-DNA-PKcs-B (400–1025 aa) WT or K525R. **b** The GFP stripes ware quantitated. The average of 10 replicates were measured and analyzed. Error bars represent standard deviation: ns indicates *P* ≥ 0.05, *P < 0.05, and **P < 0.01. **c** 293 T cells were transfected with Flag-DNA-PKcs-B (400–1025 aa) WT and K525R plasmids, the Co-IP assays were performed and samples were detected by Western Blotting. **d** GST pull-down assays were performed with GST-DNA-PKcs-B (400–1025 aa) WT or K525N protein, samples were separated by SDS-page and detected by Western Blotting. **e** GST pull-down assays were performed with purified GST-DNA-PKcs-B (400–1025 aa) WT/K525N, Ku70/Ku80 protein, samples were separated by SDS-page and detected by Western Blotting. **f** GST pull-down assays were performed with purified GST-DNA-PKcs-B (400–1025 aa) WT/K525N, Ku70 and Ku80 protein, samples were separated by SDS-page and detected by Western Blotting.

To ascertain the biological significance of K525 crotonylation on DNA-PKcs function following DNA damage, we performed laser microirradiation experiment using U2OS cells stably transfected with GFP-DNA-PKcs-B (400–1025 aa) WT or K525R mutant plasmids. The K525R mutation delayed and impaired the recruitment of GFP-DNA-PKcs-B (400–1025 aa) to DNA damage sites resulting from laser microirradiation (Fig. 3a, b).

DNA-PKcs has been reported in previous research to form a complex with KU, which is responsible for its recruitment to DNA damage sites [6]. To determine whether DNA-PKcs-B (400–1025 aa) K525 crotonylation facilitates the interaction between DNA-PKcs and KU, Co-IP assays were conducted in the absence of DNA in 293 T cells transfected with Flag-DNA-PKcs-B (400–1025 aa) WT or the K525R mutant. Interestingly, according to our results, the interaction between DNA-PKcs-B and KU was increased upon DNA damage, and K525 crotonylation did not impact this interaction (Fig. 3c). To confirm these results, GST pull-down was performed with GST-DNA-PKcs-B (400–1025 aa) WT and K525N (mock crotonylation [45]) protein and the same results were demonstrated (Fig. 3d).

Then in vitro GST-pull down assays were performed with purified GST-DNA-PKcs-B (400–1025 aa) WT/K525N, Ku70/Ku80. The results showed that DNA-PKcs K525N mutation had only a slight effect on its binding with Ku70 or Ku80 (Fig. 3e). Given that the formation of KU70/80 heterodimer is essential for DNA-PKcs recruiting DNA-PKcs to DNA damage site, we wondered whether K525 crotonylation promotes the interaction in a KU heterodimer-dependent manner. Later, we implemented GST pull-down assay in the presence of both Ku70 and Ku80, the same results were observed (Fig. 3f).

### DNA-PKcs K525 crotonylation promotes its DNA binding activity, which is essential for its interaction with Ku70/80/DNA complex, but not with DNA free Ku70, Ku80 or KU70/80 heterodimer

**Fig. 4:**
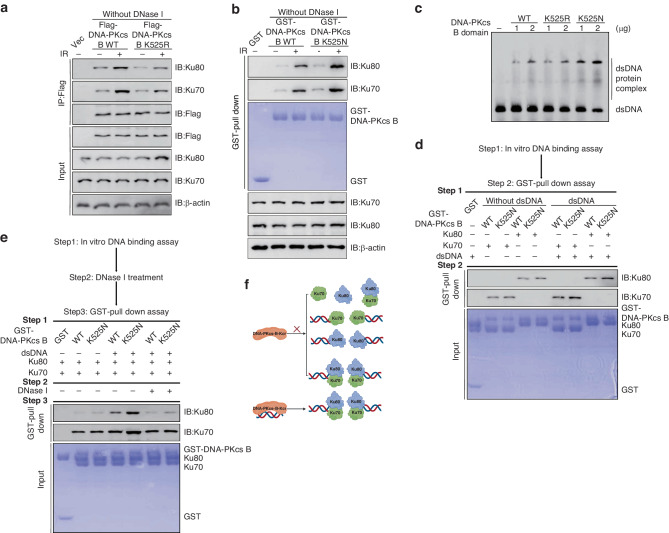
DNA-PKcs K525 crotonylation promotes its DNA binding activity, which is essential for its interaction with Ku70/80/DNA complex, but not with Ku70, Ku80 or KU70/80. **a** 293T cells were transfected with Flag-DNA-PKcs-B (400–1025 aa) WT and K525R plasmids, the Co-IP assays were performed without DNase I treatment and samples were detected by Western Blotting. **b** HeLa cells were treated with 8 Gy IR or not, and then cells were harvested and GST-pull down assays were performed with purified GST-DNA-PKcs-B (400–1025 aa) WT/K525N, samples were separated by SDS-page and detected by Western Blotting. **c** EMSA assays were performed with dsDNA and DNA-PKcs-B (400–1025 aa) WT and K525N mutant proteins. **d** GST pull-down assays were performed in the present of dsDNA by using GST-DNA-PKcs-B (400–1025 aa) WT or K525N and Ku70 or Ku80 proteins, samples were separated by SDS-page and detected by Western Blotting. **e** GST pull-down assays were performed by using GST-DNA-PKcs-B (400–1025 aa) WT or K525N in the present of dsDNA, Ku70 and Ku80 proteins, samples were separated by SDS-page and detected by Western Blotting. **f** Model of the mechanism DNA-PKcs B (400–1025 aa) domain crotonylation effects its interaction with Ku70/80/DNA complex.

Given that the K525 crotonylation site of DNA-PKcs is located in DNA-PKcs DNA binding domain, we presume that K525 crotonylation may promote the interaction between DNA-PKcs and Ku in a DNA-dependent manner. Then, Co-IP assay was performed in the present of DNA in 293 T cells transfected with Flag-DNA-PKcs-B (400–1025 aa) WT and K525R mutant. The K525R mutant attenuated the increase in the interaction between DNA-PKcs-B (400–1025 aa) and KU upon DNA damage (Fig. 4a). Additionally, the GST pull-down assay also indicated that K525 crotonylation significantly enhanced the binding between DNA-PKcs-B (400–1025 aa) and KU upon DNA damage in the present of DNA (Fig. 4b). These results demonstrate that DNA-PKcs K525 crotonylation facilitates the recruitment of DNA-PKcs to DNA damage sites by promoting its interaction with KU in a DNA-dependent manner. To determine whether K525 crotonylation promotes DNA binding activity of DNA-PKcs-B (400–1025 aa), EMSA assay was performed. We found that the crotonylation-mimic K525N mutation significantly enhanced the DNA binding ability of DNA-PKcs-B (400–1025 aa) (Fig. 4c).

To decipher the function and mechanism of DNA-PKcs DNA binding activity in the assembly of DNA-PK complex, GST pull-down assays were performed with GST-DNA-PKcs-B (400–1025 aa) WT or K525N and Ku70 or Ku80 protein in the presence or absence of DNA. Surprisingly, K525 crotonylation had a slight effect on DNA-PKcs binding to Ku70 or Ku80 molecular even in the presence of DNA (Fig. 4d). Given that the formation of KU70/80 heterodimer is essential for the recruitment of DNA-PKcs to DNA damage sites, we presume DNA-PKcs K525 crotonylation may promote its binding to the Ku70/80/DNA complex, subsequently, GST pull-down assays were performed. K525N mutation significantly enhanced the interaction between DNA-PKcs-B (400–1025 aa) and KU in the presence of DNA, which was abolished by DNase I treatment (Fig. 4e). Our results demonstrate that DNA-PKcs K525 crotonylation facilitates its binding to Ku70/80/DNA complex, either Ku70, Ku80, Ku70/Ku80, Ku70/DNA or Ku80/DNA (Fig. 4f). Our results also indicate that DNA-PKcs K525 crotonylation is essential for the formation of DNA-PK complex.

### DNA-PKcs K525 crotonylation facilitates the assembly of the DNA-PK complex

**Fig. 5:**
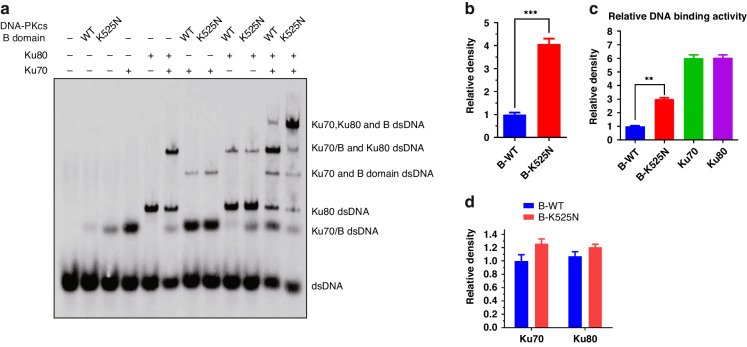
DNA-PKcs K525 crotonylation facilitates the assembly of DNA-PK complex. **a** DNA-PK assembly assay was performed by using DNA-PKcs-B (400–1025 aa) WT/K525N and Ku70, Ku80 protein according to the procedures of EpiMark® Nucleosome Assembly Kit. **b** Quantitated of Lanes 11,12. The average of 3 replicates were measured and analyzed. Error bars represent standard deviation: ***P < 0.001. **c** Quantitated of Lanes 2, 3, 4, and 5. The average of 3 replicates were measured and analyzed. **P < 0.01. **d** Quantitated of Lanes 7, 8, 9, and 10. The average of 3 replicates were measured and analyzed.

The above results demonstrated that DNA-PKcs DNA binding status is essential for its binding to the Ku70/80/DNA complex and is pivotal for the assembly of DNA-PK complex. To assess the impact of DNA-PKcs K525 crotonylation on DNA-PK complex assembly, in vitro DNA-PK assembly assays were conducted using Biotin-dsDNA, DNA-PKcs-B (400–1025 aa), Ku70, and Ku80. The results indicated that the DNA-PKcs K525N (crotonylation mimic) mutation, significantly enhances the assembly of DNA-PK complex (Fig. 5a lanes11,12 and Fig. 5b). Our results also demonstrated that the DNA binding activity of Ku70 and Ku80 was much higher than DNA-PKcs B (400–1025 aa) domain, even DNA-PKcs-B (400–1025 aa) crotonylation status (Fig. 5a lanes 2, 3, 4, 5 and Fig. 5c). This result explains why Ku70/80 are more effective than DNA-PKcs at binding to DSBs sites upon DNA damage. In addition, our result showed that DNA-PKcs-B (400–1025 aa) WT and K525N/DNA complex had no bias in its binding to Ku70/DNA and Ku80/DNA complex (Fig. 5a lanes 7, 8, 9, 10 and Fig. 5d), which was consistent with our above results showing that DNA-PKcs crotonylation promoted its binding to Ku70/80/DNA complex, either Ku70/DNA or Ku80/DNA complex.

### GCN5-mediated DNA-PKcs crotonylation promotes DNA-PK assembly, DNA-PKcs recruitment and signal transduction upon DNA damage

**Fig. 6:**
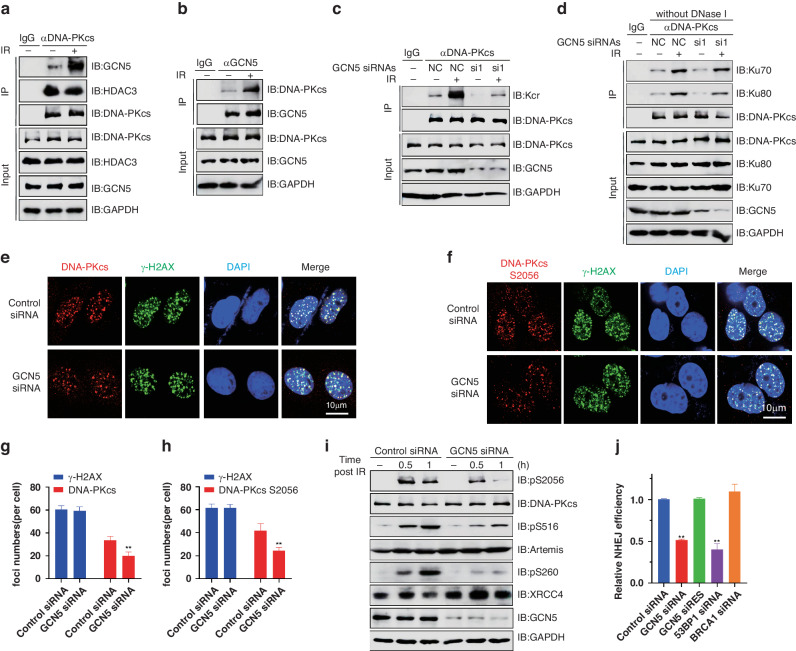
GCN5-mediated DNA-PKcs crotonylation promotes DNA-PK assembly, DNA-PKcs recruitment and activation upon DNA damage. **a**, **b** HeLa cells were treated with IR or not, then Co-IP assays were performed with indicated antibodies and samples were separated by SDS-page and detected by Western Blotting. **c** HeLa cells were transfected with indicated siRNA and treated with IR or not, then denature IP was performed and DNA-PKcs crotonylation was analyzed by Western blotting. **d** HeLa cells were transfected with indicated siRNA and treated with IR or not, then Co-IP was performed with anti-DNA-PKcs antibody, samples were separated by SDS-page and detected by Western blotting. **e**, **g** HeLa cells were transfected with indicated siRNA and treated with IR, then immunofluorescent (IF) staining assay was performed on DNA-PKcs and γH2AX foci. Quantification of DNA-PKcs foci were measured and 50 cells were analyzed via IF staining assay for DNA-PKcs and γH2AX. **P < 0.01. **f**, **h** HeLa cells were transfected with indicated siRNA and treated with IR or not, then immunofluorescent (IF) staining assay of DNA-PKcs pS2056 and γH2AX foci was performed (L). Quantification of DNA-PKcs pS2056 foci were measured and 50 cells were analyzed(K). **P < 0.01. **i** HeLa cells were transfected with indicated siRNA and treated with or without IR, then cells were harvested and the levels of DNA-PKcs pS2056(M), Artemis pS516 and XRCC4 pS260(N) were detected by Western Blotting. **j** The NHEJ efficiency was determined using the EJ5-GFP reporter assay. BRCA1 or 53BP1 siRNAs were used as a positive or negative control, respectively. Data are means ± SD from three independent experiments. **P < 0.01.

Given that radiation exposure increases the crotonylation of DNA-PKcs, the interaction of DNA-PKcs with the crotonylation mediators GCN5 or HDAC3 may be influenced by irradiation. The Co-IP assays showed that the interaction between DNA-PKcs and GCN5, but not HDAC3, was dramatically increased after the irradiation (Fig. 6a, b). Additionally, knockdown of GCN5 attenuated the IR-induced increase in DNA-PKcs crotonylation (Fig. 6c). Hence, GCN5 is associated with the DNA damage-induced increasement of DNA-PKcs crotonylation.

To assess the function of GCN5 in DNA-PK assembly, Co-IP assays were conducted without DNase treatment. Knockdown of GCN5 dramatically attenuated the increase in DNA-PKcs binding to KU induced by DNA damage (Fig. 6d). Considering that DNA-PKcs binding to KU is crucial for DNA-PKcs recruitment to DNA damage sites. DNA-PKcs foci were detected in GCN5-knockdown cells, and the results showed that GCN5-knockdown significantly reduced the number of DNA-PKcs foci upon irradiation (Fig. 6e, g). Additionally, by using laser microirradiation experiments, we found that GCN5 deficiency had no effect on the recruitment of GFP-Ku70 or GFP-Ku80 to DNA damage sites (Fig. S2A–D).

Given that DNA-PKcs interacts with KU and recruitment to sites of DNA damage is pivotal for its kinase activation, then DNA-PKcs pS2056, which is a marker of its kinase activity of autophosphorylation, was detected. As depicted in Fig. 6f, h, knocking down GCN5 inhibits the formation of DNA-PKcs pS2056 foci. Western blotting analysis further confirmed that knockdown of GCN5 dramatically attenuated the phosphorylation level of DNA-PKcs at the S2056 site (Fig. 6i). We further determined the influence of GCN5 on the downstream substrates of DNA-PK-dependent signaling and found that knockdown of GCN5 dramatically reduced the DNA damage-induced phosphorylation of Artemis pS516 and XRCC4 pS260 (Fig. 6i). Given the necessity of DNA-PK for DSBs repair via NHEJ pathway, then NHEJ assay were subsequently performed. GCN5 deficiency dramatically impaired NHEJ pathway (Fig. 6j). Clearly, GCN5-mediated DNA-PKcs K525 crotonylation plays a crucial role in DNA-PK assembly and activation, as well as in the recruitment of DNA-PKcs to DNA damage sites in vivo.

### GCN5 deficiency impairs DNA repair and exacerbates the outcomes of DNA damaging agents in cancer therapy

**Fig. 7:**
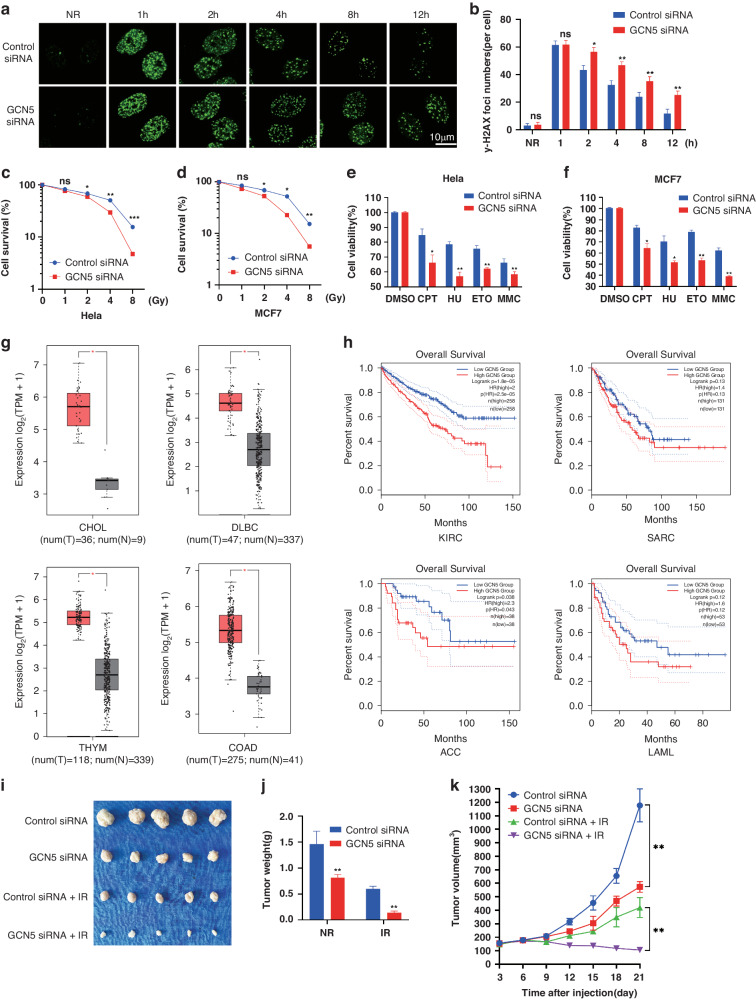
GCN5 deficiency impairs DNA repair and exacerbates the outcomes of DNA damaging agents in cancer therapy. **a**, **b** HeLa cells were transfected with indicated siRNA and treated with IR or not, then IF assay were performed with γ-H2AX antibody. Focis in 50 cells were counted for quantification (**b**). *P < 0.05, **P < 0.01. HeLa (**c**) and MCF-7 (**d**) cells were transfected with indicated siRNA, then formation assays were performed. Data represent the mean ± SD for triplicate experiments. *P < 0.05, **P < 0.01. Sensitivity of GNC5 depleted HeLa (**e**) and MCF-7 (**f**) cells to DNA damage or replication stress–inducing agents was determined by MTS assays. Data are means ± SD from three biological triplicates. *P < 0.05, **P < 0.01. **g** GCN5 is expressed differentially in varying carcinoma samples and normal tissue controls. CHOL Cholangiocarcinoma, lymphoid neoplasm, DLBC diffuse large B cell lymphoma, COAD Colon adenocarcinoma and thymoma (THYM). **h** The overall survival analysis of GCN5 expression in different tumor patients. KIRC: Kidney renal clear cell carcinoma, SARC Sarcoma, ACC Adrenocortical carcinoma, LAML Acute Myeloid Leukemia. **i**–**k** Tumorigenicity of GCN5 WT and GCN5 knock down HeLa cells in nude mice. GCN5 WT (0.1 ml; 1 × 10^7^ cells) or GCN5 knock down HeLa cells were used in a xenograft tumor assay with 8 Gy γ-ray irradiation treatment or not, and tumor weight and tumor volume were quantified. Data are means ± SD. ns indicates *P* ≥ 0.05. **P < 0.01.

Given that GCN5 deficiency results in the attenuation of DNA-PKcs signaling, which is essential for DNA repair through the NHEJ pathway, the functions of GCN5 in DNA repair were investigated. IF assays were performed to examine γH2AX, a specific marker of DNA DSBs. As shown in Fig. 7a, b, GCN5 deficiency led to an increase in the number of residual γH2AX foci at least 2 h post IR. γH2AX levels were also examined through Western Blotting, and the results showed that knockdown of GCN5 resulted in an increase in γH2AX (Fig. S3A). We investigated whether GCN5 knockdown can prompt cancer cell death post irradiation. GCN5 deficiency increased dramatically increased the induction of cancer cell apoptosis induced by IR (Fig. S3B, C). As confirmed by colony formation assays on HeLa (Fig. 7c) and MCF7 (Fig. 7d) cells, cancer cells deficient in GCN5 exhibited extremely sensitive to IR. Furthermore, GCN5 deficiency increased the sensitivity of HeLa (Fig. 7e) and MCF7 cells (Fig. 7f) to additional DNA-damaging drugs, including hydroxyurea (HU), Camptothecin (CPT), Etoposide (ETO), and Mitomycin C (MMC). These findings suggest that GCN5 could potentially serve as a target for cancer therapy.

To determine whether GCN5 levels are associated with the development of carcinoma in patients. We analysed the mRNA-seq dataset from TCGA tumors, which included CHOL, DLBC, THYM and COAD. According to the abovementioned four datasets, GCN5 was upregulated in tumor tissues compared with noncarcinoma tissues (Fig. 7g). Furthermore, higher GCN5 expression within cancer samples was evidently associated with poor overall survival (OS) in KIRC, SARC, ACC, and LAML malignancies, as demonstrated by Kaplan–Meier analysis (Fig. 7h). Finally, we performed the tumor growth assay by using tumor-harboring nude mice. According to our results, compared with those of the GCN5-WT counterpart, the growth of tumors from GCN5-knockdown cancer cells was markedly suppressed after irradiation (Fig. 7i–k).

## Discussion

**Fig. 8:**
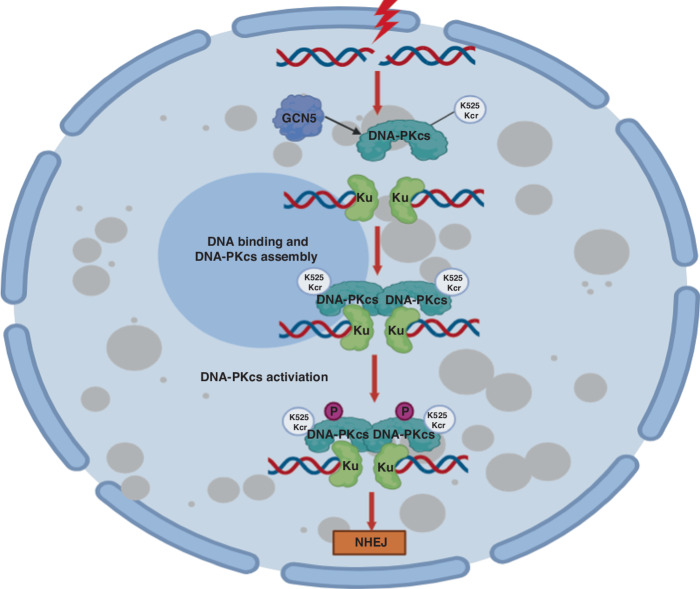
GCN5 crotonylates DNA-PKcs to promotes DNA-PKcs kinase activity and DNA double-strand break repair via NHEJ repair. Upon DNA damage, GCN5 crotonylates DNA-PKcs at K525 site, which promotes DNA-PKcs DNA binding activity and DNA-PK assembly. DNA-PKcs K525 crotonylation facilitates the DNA-PK assembly through promoting DNA-PKcs/DNA binding with Ku70/80/DNA complex, and leads to activation of DNA-PK and NHEJ repair.

The presence of DNA plays a crucial role in the maintain DNA-PK stability and activation, but the precise molecular regulatory mechanism and functions of DNA-PKcs DNA binding activity in this process is remain unclear. This study elucidates a critical regulatory process involving DNA-PK complex assembly and activation through GCN5-mediated crotonylation of DNA-PKcs, which promotes DNA-PKcs binding to DNA and its interaction with Ku70/80/DNA complex, thereby facilitating DSB repair initiation of NHEJ pathway. Our findings unveil a new mechanism governing the interactions between DNA-PKcs, DNA and Ku70/80 to assemble into the active DNA-PK complex for initiating DNA DSBs repair (Fig. 8). Our study revealed that the histone acetyltransferase GCN5 function as crotonyltransferease and acts as an optimistic new component of NHEJ pathway for DSB repair by interacting with and crotonylating DNA-PKcs upon DNA damage (Fig. 8). GCN5 deficiency results in impaired DNA-PK activation and NHEJ, sensitizing cancer cells to DNA damage induction. Therefore, our work suggested that targeting DNA-PKcs DNA-binding activity and suppressing GCN5 could be could be candidate approaches for treating tumors in combination with DSB-inducing agents.

The assembly and activation of DNA-PK complex are DNA-dependent [46] on DNA, and they are intricately regulated by multiple post-translation modifications on its components, including DNA-PKcs and Ku70/80 [14, 23, 24]. SIRT6, a deacetylase, dynamically associates with chromatin to facilitate the mobilization of DNA-PKcs onto chromatin following DNA damage. It also stabilizes DNA-dependent protein kinase (DNA-PK) at DSBs [23]. SIRT2 can also interact with DNA-PKcs to facilitate DNA-PKcs interaction with Ku and subsequent localization to DSBs [24]. Here, we revealed that the DNA-PKcs K525 residue located in the DNA-binding domain is a critical site for crotonylation and is a factor that positively regulates DNA-PKcs DNA-binding activity and DNA-PKcs assembly. The interaction between DNA-PKcs and Ku plays an important role in DNA-PK assembly. DNA-PKcs interfaces with Ku at multiple sites within its N-HEAT (1–872 aa) and M-HEAT domains (890–2580 aa). As mentioned above, SIRT2-mediated DNA-PKcs deacetylation promotes the interaction between DNA-PKcs and KU70/80 heterodimer, as well as the assembly of DNA-PK [24]. Previous studies have shown that direct binding of DNA-PK to DNA facilitates the activation of DNA-PK, and in vitro, DNA-PKcs can bind directly to DNA in a manner of independent of KU dimer [9, 47]. However, the regulatory mechanism of the DNA-PKcs DNA-binding activity and its role in the assembly and activation of DNA-PK following DNA damage is still unclear. Building upon the discovery of GCN5’s crotonylation activity towards DNA-PKcs, we further reveal that DNA damage increases the interaction between GCN5 and DNA-PKcs. This interaction led to the elevation of DNA-PKcs K525 crotonylation, subsequently facilitating DNA-PKcs binding to DNA and the formation and activation of DNA-PK. Notably, although HDAC3 has been identified as the decrotonylase for DNA-PKcs, the DNA damage induced by ionizing radiation does not appear to influence the interaction between HDAC3 and DNA-PKcs.

The C-terminal of Ku80 has been reported to function in DNA-PKcs recruitment [48]. Multiple studies have also demonstrated that Ku70, the formation of KU70/80 heterodimer and DNA, also has important effects on DNA-PKcs recruitment and DNA-PK assembly [10, 46, 49, 50]. However, the regulatory mechanism how Ku70/K80 interacts with DNA-PKcs and facilitates its recruitment is still unclear. Our study revealed that the DNA-PKcs-DNA binding status facilitates its binding to Ku70/80/DNA, either Ku70, Ku80, Ku70/80, Ku70/DNA or Ku80/DNA, which are dependent on crotonylated DNA-PKcs. Hence, our study elucidates why the formation of KU70/80 heterodimer and its binding to DSBs are prerequisites for DNA-PKcs recruitment and DNA-PK assembly. Our study also demonstrated that DNA-PKcs recruitment to DSBs sites is regulated not only regulated by protein-protein interactions but also by DNA. DNA-PKcs recruitment and DNA-PK assembly are manipulated by multiple factors, the function of other DNA-PKcs sites and domains in the regulation of these processes need further research.

For decades, that DNA-PKcs was shown to possess DNA binding capability, and its kinase activity requires DNA. As indicated by the DNA-PKcs structure and the findings of several studies, autophosphorylation of the DNA-PKcs ABCDE cluster allosterically loosens it from the DNA end. DNA-PK can tighten its DNA-end binding in the inactive state with ABCDE [14, 51–53]. Nevertheless, the regulatory mechanism governing its DNA binding activity in response to DNA damage is remains unclear. Our study revealed that GCN5-mediated DNA-PKcs K525 crotonylation, which occurs in the DNA binding domain, promotes its DNA binding activity and activation. DNA-PKcs kinase activation is subject to regulation through various PTMs. In our previous reports, we highlighted the significance of PARP-1 mediated PARylation and HUWE1-mediated neddylation in DNA-PKcs autophosphorylation and activation [21, 22]. In the current study, we unveil the role of GCN5 in crotonylating and activating DNA-PKcs in response to DNA damage. DNA-PKcs crotonylation promotes its binding to DNA and localizing to DSB sites.

Protein crotonylation is emerging as an important player in the regulation of DNA damage signal transduction. Crotonylation generally regulates the functions of DNA repair proteins by modulating protein-protein interactions and DNA binding activities [54]. For example, RPA1 crotonylation promotes its ssDNA binding ability to facilitating RPA1 recruitment to sites of DNA damage and HR repair [29]. Crotonylation serves as a switchboard to determine DNA repair pathway choice. Previous reports have indicated that the DNA mismatch repair protein MSH6 can be crotonylated, which is negatively regulated by dibutyl phthalate. Mutation of the crotonylation site K544 on MSH6 impairs its association with Ku70, leading to enhanced NHEJ while inhibiting HR [55]. In our study, we demonstrated that DNA-PKcs crotonylation plays a role in regulating both DNA-PKcs DNA binding activity and its interaction with KU70/80 heterodimer. We propose that one of the significant functions of crotonylation in DNA repair is to modulate the DNA-binding activity of repair proteins.

Being one of the most extensively studied acetyltransferases, GCN5 exerts a critical influence on DNA damage repair and gene transcription by regulating the acetylation of H3 and SWI/SNF [44, 56, 57]. It has also been reported that DNA-PKcs phosphorylates GCN5 and attenuates its acetyltransferase activity [58, 59], and GCN5 has also been reported to be a crotonyltransferase that mediates histone crotonylation [28, 60]. Here, we showed that GCN5 can also act as a crotonyltransferase non-histone, and the DNA-PKcs K525 site is its substrate. The study also shows that GCN5 interacts more with DNA-PKcs after DNA damage caused by IR. However, the mechanism by which IR exposure increases this interaction is remains unclear. Nevertheless, DNA-PKcs K525 is located in the N-terminal HEAT repeat domain of DNA-PKcs and is related to the interactions with Ku70/80 and DNA-binding. Our results clearly indicate that crotonylation of DNA-PKcs K525 plays an essential role in the interaction of DNA-PKcs with Ku70/80 and recruitment to DNA damage sites. GCN5 deficiency impairs DNA-PKcs recruitment, the formation and activation of DNA-PK complex, thereby decreasing DSBs NHEJ repair efficiency. GCN5 deficiency also led to attenuation of G1/S arrest (Fig. S4) and tumor cells sensitivity to DNA damage agents. Considering the interplay between DNA-PK and GCN5 and the acetylation of DNA-PKcs by TIP60 during DNA damage stress, it is presumed that there may be a modulation between acetylation and crotonylation of DNA-PKcs to dynamically control its roles in DNA damage response. Clearly, GCN5 emerges as a novel regulator for the repair of DNA double-strand breaks, and its dysfunction could result in genomic instability and alterations in cellular sensitivity to DNA damage agents such as ionizing radiation.

In summary, we have identified a novel target for crotonylation involved in DNA damage repair. We have also elucidated a regulatory mechanism for DNA-PKcs DNA binding activity, DNA-PK assembly, and activation by GCN5-mediated DNA-PKcs K525 crotonylation. This discovery represents a critical initial signaling event that initiates the NHEJ repair pathway, contributing to the preservation of genome integrity (Fig. 8). Additionally, our study offers a mechanistic basis and preclinical groundwork for the rational targeting of GCN5 and the DNA-binding activity of DNA-PKcs. This approach holds promise as a potential treatment strategy to enhance outcomes in cancer-specific radiotherapy and DNA damage-inducing chemotherapy.

### Supplementary information

supplementary

## Data Availability

The data in this manuscript is available from the corresponding author upon request.
