# Supplementary material for: GCN5 mediates DNA-PKcs crotonylation for DNA double-strand break repair and determining cancer radiosensitivity
Source: Br J Cancer. 2024 Apr 4;130(10):1621–34. doi: 10.1038/s41416-024-02636-4 (PMC11091118; doi:10.1038/s41416-024-02636-4)
Supplement: Supplementary file 1 — supplementary [file 41416_2024_2636_MOESM1_ESM.pdf]

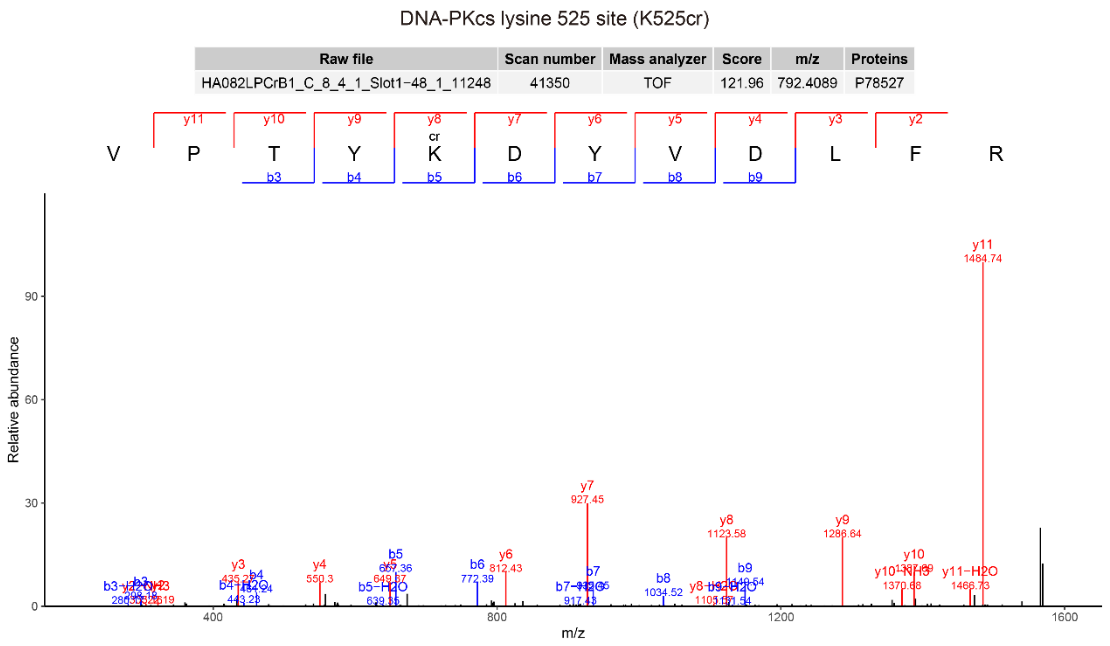

Figure S1 Identification of crotonylation sites in DNA-PKcs by high resolution LC-MS/MS

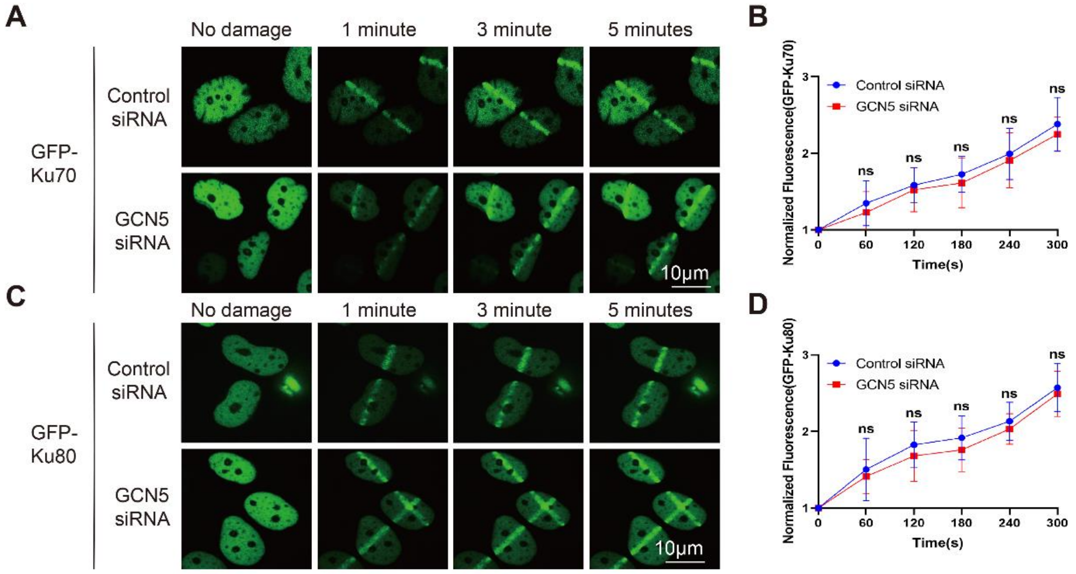

Figure S2 GCN5-mediated DNA-PKcs crotonylation does not affect Ku70/80 recruitment at the damage site

(A, B, Cand D) HeLa cells transfected with GFP-Ku70(A), GFP-Ku80(C) and indicated siRNA were subjected to laser microirradiation. The laser

output was set to 7.5%. Fluorescence of the GFP stripes was measured for GFP-Ku70 (B) and GFP-Ku80 (D) and analyzed by using Image J Software. The average of 10 replicates at each time point per condition was plotted. ns indicates $P \geq 0.05$.

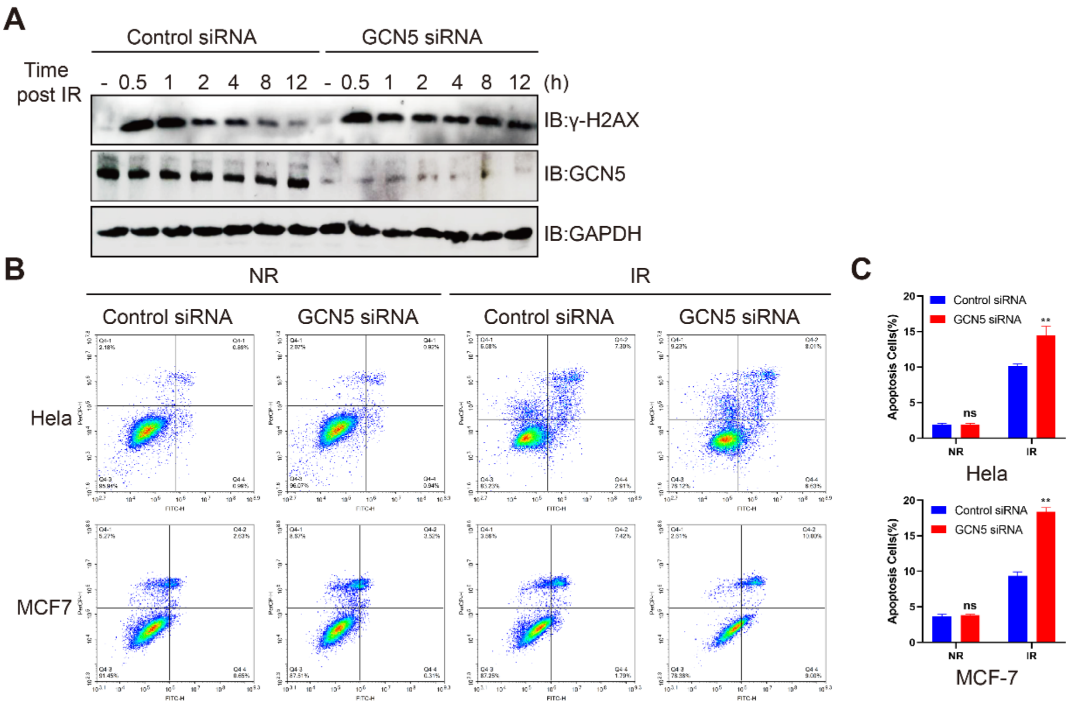

Figure S3 GCN5 knockdown causes increased DNA double-strand breaks and apoptosis

(A) HeLa cells were transfected with indicated siRNA and treated with IR or not, then cells were harvested and detected by Western Blotting.

(B and C) GCN5 knockdown HeLa and MCF-7 cells were treated with 8 Gy γ-ray irradiation or not. Apoptosis was detected at 24 hours after treatments. Quantification (C) of apoptosis induction. Data are means ± SD from three biological triplicates. ns indicates $P \geq 0.05$, $^{**}P < 0.01$.

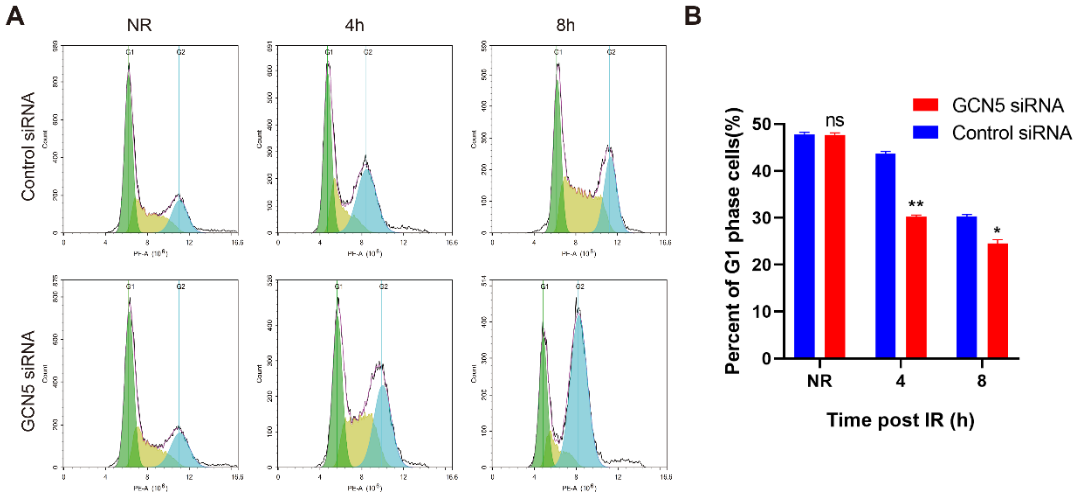

Figure S4 GCN5 deficiency leads to attenuation of G1/S arrestment

(A) HeLa cells were transfected with indicated siRNA and treated with 8 Gy γ-ray irradiation or not. The G1 phase cells was measured at different time points after IR by Flow cytometry.

(B) Quantification of the G1 phase cells induction. Data are means ± SD from three biological triplicates. ns indicates $P \geq 0.05$, $*P < 0.05$, $**P < 0.01$.
